# *In vitro* amplification of ovine prions from scrapie-infected sheep from Great Britain reveals distinct patterns of propagation

**DOI:** 10.1186/1746-6148-8-223

**Published:** 2012-11-15

**Authors:** Leigh Thorne, Thomas Holder, Andrew Ramsay, Jane Edwards, Maged Mohamed Taema, Otto Windl, Ben Charles Maddison, Kevin Christopher Gough, Linda Ann Terry

**Affiliations:** 1 Animal Health Veterinary Laboratories Agency (AHVLA), Woodham Lane, New Haw, Addlestone, Surrey, KT15 3NB, UK; 2 School of Veterinary Medicine and Science, The University of Nottingham, Sutton Bonington Campus, College Road, Sutton Bonington, Leicestershire, LE12 5RD, UK; 3 ADAS UK, School of Veterinary Medicine and Science, The University of Nottingham, Sutton Bonington Campus, College Road, Sutton Bonington, Leicestershire, LE12 5RD, UK; 4 Current address: Department of Immunology and Pathology, CMU, University of Geneva, 1 Rue Michel Servet, Geneva 4, 1211, Switzerland

**Keywords:** Prions, Transmissible spongiform encephalopathy, Scrapie, Sheep, PMCA

## Abstract

**Background:**

Protein misfolding cyclic amplification (PMCA) is a method that facilitates the detection of prions from many sources of transmissible spongiform encephalopathy (TSE). Sheep scrapie represents a unique diversity of prion disease agents in a range of susceptible *PRNP* genotypes. In this study PMCA was assessed on a range of Great Britain (GB) sheep scrapie isolates to determine the applicability to veterinary diagnosis of ovine TSE.

**Results:**

PrP^Sc^ amplification by protein misfolding cyclic amplification (PMCA) was assessed as a diagnostic tool for field cases of scrapie. The technique was initially applied to thirty-seven isolates of scrapie from diverse geographical locations around GB, and involved sheep of various breeds and *PRNP* genotypes. All samples were amplified in either VRQ and/or ARQ PrP^C^ substrate. For PrP^Sc^ from sheep with at least one VRQ allele, all samples amplified efficiently in VRQ PrP^C^ but only PrP^Sc^ from ARH/VRQ sheep amplified in both substrates. PrP^Sc^ from ARQ/ARQ sheep displayed two amplification patterns, one that amplified in both substrates and one that only amplified in ARQ PrP^C^. These amplification patterns were consistent for a further 14/15 flock/farm mates of these sheep. Furthermore experimental scrapie strains SSBP1, Dawson, CH1641 and MRI were analysed. SSBP1 and Dawson (from VRQ/VRQ sheep) amplified in VRQ but not ARQ substrate. MRI scrapie (from ARQ/ARQ sheep) nor CH1641 did not amplify in ARQ or VRQ substrate; these strains required an enhanced PMCA method incorporating polyadenylic acid (poly(A)) to achieve amplification.

**Conclusions:**

PrP^sc^ from 52 classical scrapie GB field isolates amplified in VRQ or ARQ or both substrates and supports the use of PMCA as a rapid assay for the detection of a wide range of ovine classical scrapie infections involving multiple *PRNP* genotypes and scrapie strains.

## Background

Transmissible spongiform encephalopathies (TSEs) or prion diseases are a group of fatal neurodegenerative disorders which include Creutzfeldt–Jakob disease (CJD) and Kuru in humans, bovine spongiform encephalopathy (BSE) in cattle, scrapie in sheep and goats and chronic wasting disease (CWD) in deer and elk. Experimental evidence supports the concept that the causative agent is comprised of a misfolded isoform (PrP^Sc^) of the normal, ubiquitously expressed cellular protein, PrP^C^[1]. The two proteins are of identical primary sequence and share the same posttranslational modifications yet are distinct in their physicochemical properties. The detection of accumulated PrP^Sc^ following post-mortem is a reliable marker for the presence of infection and forms the basis of the majority of diagnostic tests. Currently there is no cure or treatment for these diseases and the development of high throughput diagnostics to detect disease prior to the onset of clinical signs are still required. To date there are no validated blood tests for sheep scrapie and the most promising pre-mortem tests involve biopsy of lymphoreticular tissue and immunohistochemical (IHC) examination [2,3].

Conversion of PrP^C^*in vivo* is presumed to occur following direct interaction with the pathogenic protein, which acts as a template to drive the conformational change to PrP^Sc^. Sustained propagation, largely in the central nervous system, results in the accumulation and deposition of the pathogenic protein. This conversion can be reproduced *in vitro* using the technique protein misfolding cyclic amplification (PMCA) which was pioneered by Soto and colleagues [4]. PMCA allows propagation of PrP^Sc^*in vitro* from very small amounts of undetectable seeding material to quantities sufficient for detection by Western blot or plate-based immunoassays. This ultra-sensitive method has been previously applied to identify prions in a wide range of tissues and fluids from scrapie-infected sheep (blood, faeces, saliva and milk) where only small amounts of the infectious agent reside [5-9]. Given its unique ability to detect prions in readily accessible tissues and at preclinical stages of the disease it presents a viable method for a live animal, preclinical test for prion disease.

The application of PMCA to detect PrP^Sc^ from infected sheep had previously been focused on infected sheep with highly susceptible genotypes and from a single endemic scrapie infected flock. The data from these studies showed that pre-mortem and pre-clinical testing for prion diseases could be achieved [5-9]. However, these studies were not representative of the diversity of scrapie strains or *PRNP* genotypes observed in sheep. Within the present study we aimed to determine whether scrapie from diverse locations and *PRNP* genotypes in Great Britain (GB) could be amplified *in vitro* and therefore whether PMCA could be used universally as a putative diagnostic tool for a wide range of primary isolates of classical scrapie.

## Results

### Amplification of classical scrapie field isolates by PMCA

Prions derived from brain tissue from sheep infected with classical scrapie of 8 different *PRNP* genotypes (at codons 136, 141 and 154) were used to seed PMCA reactions. These amplifications used either VRQ homozygous and/or ARQ homozygous brain substrates from prion-free sheep as a source of PrP^C^ for conversion. A total of 37 sheep were initially tested from diverse geographical regions of GB and from at least 20 different farms (Table 1). All field cases were from 1997–2007 and were confirmed as scrapie-positive by three independent diagnostic tests; the Bio-Rad TeSeE immunoassay, the hybrid Western Blot developed at the AHVLA [10] and immunohistochemistry (IHC) of formalin-fixed paraffin-embedded sections. All of these cases displayed characteristics of classical scrapie when analysed by Western blot and IHC (as reported for statutory diagnosis, data not shown).

**Table 1:** ***In vitro *PMCA amplification of sources of GB scrapie**

**Sheep ID**	**PrnP genotype**	**Farm**	Geographic Location^*^	**Age at death**	**Amplification in substrates**	
					**VRQ**	**ARQ**
1597/98	VRQ/VRQ	1	E. England	2y 7 m	+	-
322/97		2	Wales	2y 0 m	+	-
1563/02		3	W. England	2y 8 m	+	-
836/03		4	S. England	2y 2 m	+	-
1276/02		5	Wales	2y 0 m	+	ND^#^
2413/98		6	W. England	2y 2 m	+	ND
456/03	ARQ/VRQ	7	N. England	3y 0 m	+	-
455/03		7	N. England	6y 0 m	+	-
615/03		7	N. England	5 y 0 m	+	-
226/03		7	N. England	5y 0 m	+	-
ss2-022278		8	W. England	not known	+	-
219/06	ARH/VRQ	17	W. England	2y 0 m	+	+
252/06		17	W. England	2y 0 m	+	+
198/06		17	W. England	2y 0 m	+	+
216/06		17	W. England	2y 0 m	+	+
989/02	ARR/VRQ	9	S. England	8y 0 m	+	ND
ss4 506045		10	N. England	not known	+	-
ss4-506228		not known	not known	not known	+	-
297/97	ARQ/ARQ	11	S. England	not known	-	+
ss4-012265		10	N. England	not known	-	+
913/05		12	W. England	5y 0 m	+	+
522		not known	not known	not known	ND	+
960/06		13	W. England	7y 0 m	+	+
1341/04		14	E. England	5y 5 m	-	+
1230/04		15	Scotland	2y 4 m	+	+
284/97	AHQ/AHQ	11	S. England	not known	-	+
1091/01		16	Midlands	4y 8 m	-	+
217/06	ARH/ARH	17	W. England	1y 0 m	+	+
429/06		17	W. England	2y 0 m	+	+
1731/03	ARH/ARQ	18	N. England	1y 6 m	+	+
1741/03		18	N. England	1y 2 m	+	+
1952/04		19	W. England	3y 0 m	+	+
936/05		20	Wales	4y 0 m	+	+
708/02		15	Scotland	5y 0 m	+	+
399/04		15	Scotland	3y 0 m	+	+
462/04		15	Scotland	2y 0 m	+	+
915/07		13	S. England	1y 6 m	+	+

^*^S. England: South of England, *N. England*: North of England, *W. England*: Welsh borders and West Country, *E. England*: East of England. All samples were analysed in duplicate after four rounds of PMCA and those marked + were positive in at least one replicate. ^*#*^*ND*, not done.

All 37 samples amplified *in vitro* by serial PMCA in either the VRQ or the ARQ substrate or both (Table 1) after 4 rounds. Samples from sheep possessing at least one VRQ allele (18 isolates) amplified in a VRQ substrate. Furthermore, with the exception of ARH/VRQ sheep, none of these samples amplified in an ARQ homozygous substrate (11 out of 15 isolates tested). This observation indicates that homology for the amino acid at position 136 between seed and substrate may be required for the amplification of some isolates as previously suggested [9]. The ARH/VRQ infected sheep, PrP^Sc^ amplified in both VRQ and ARQ homozygous substrates (4 isolates). It is unclear whether this is related to the presence of the ARH allele or the identity of the isolate, given that these samples all came from the same farm. However, the effects of the ARH genotype appears the more likely explanation as the presence of the ARH allele (14 isolates) promoted amplification of PrP^Sc^ in both VRQ and ARQ substrates irrespective of the presence of a heterologous allele. This included ARH samples from geographically distinct farms.

Samples from seven ARQ/ARQ sheep were tested and all amplified readily in the ARQ substrate in agreement with previous observations [9]. However, when the ARQ/ARQ scrapie samples were tested in the VRQ substrate, heterogeneity of amplification was observed. Three out of six samples were also amplified in VRQ substrate. These samples were evaluated several times in order to verify this heterogeneity. No variation in the amplification pattern was seen when three PMCA substrates of the same genotype but from different sheep were used. The farms from which the two patterns emerged were not geographically close. The two AHQ/AHQ samples tested amplified only in the ARQ substrate.

**Figure 1:**
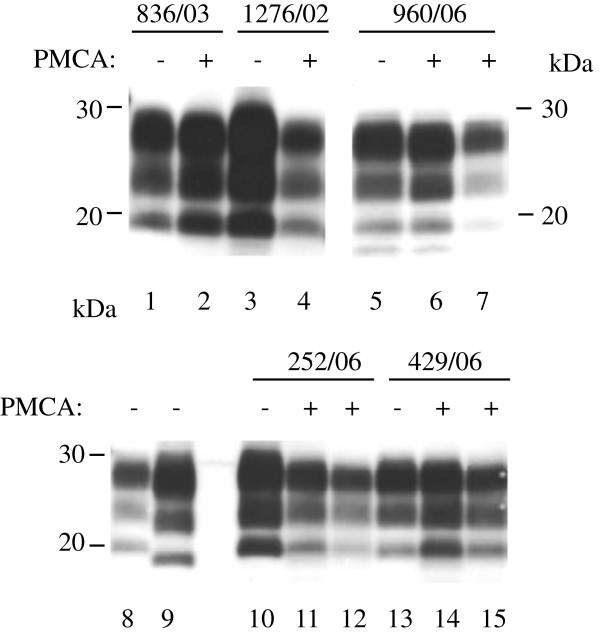
**Amplification by *in vitro *PMCA of field sources of scrapie of sheep with different *PRNP *genotypes.** Unamplified samples (−) or amplified for 4 rounds of PMCA (+) were analysed by Western blot. VRQ/VRQ scrapie 836/03 (lanes 1 and 2) and 1276/02 (lanes 3 and 4) and ARQ/ARQ scrapie 960/06 (lanes 5–7), ARH/VRQ scrapie 252/06 (lane 10–12), ARH/ARH 429/06 scrapie (lanes 13–15) were amplified in VRQ/VRQ substrate (lanes 2, 4, 6, 11 and 14) or ARQ/ARQ substrate (lane 7,12 and 15). Lanes 8 and 9: unamplified controls of classical scrapie (lane 8) and experimental ovine BSE (lane 9). PrP was visualised with Sha31 antibody. Molecular mass markers are indicated (kDa). In all lanes wet tissue equivalent of 0.3 mg was loaded.

Western blot analysis was performed on selected samples and showed that the molecular mass profiles remained unchanged following amplification of classical scrapie isolates of distinct *PRNP* genotypes in either ARQ or VRQ homozygous substrate (Figure 1). The distinct three-band pattern corresponding to the di-, mono- and un- glycosylated PK resistant PrP^sc^ moieties were clearly observed and the molecular mass of the un-glycosylated bands were indistinguishable.

The distinct patterns of amplification observed for the ARQ/ARQ seeds, i.e. ability to amplify in VRQ and ARQ or ARQ alone, were investigated further. Four farms were identified where additional samples were available; three for which the ARQ/ARQ scrapie samples amplified in both ARQ and VRQ substrates (913/05, 1230/04 and 960/06) and one farm where the original tested isolate amplified in ARQ substrates alone (297/97). Interestingly, 14 of 15 additional scrapie infected farm/flock mates tested maintained the amplification profile of the initial cases (Table 2). The sheep were from geographically distant farms and from seven different sheep breeds. Furthermore, flock/farm mates carried prions with similar *in vitro* amplification characteristics. There was one exception to this observation. Isolate 210/03 amplified in both substrates whereas the other 7 sheep tested from this farm only amplified in the ARQ substrate. Moreover, upon further analysis of sheep from this farm, two scrapie infected ARQ/VRQ sheep (1684/97 and 1927/99) produced prion that was amplified in ARQ but not VRQ substrate. This is different from the pattern of amplification that we observed for ARQ/VRQ scrapie sources as shown in Table 1.

**Table 2:** PMCA-amplification of infected flock/farm mates of ARQ/ARQ scrapie-infected sheep

Farm^*^	**Sheep ID**	**Breed**	**Age at death**	**PMCA substrates**^**#**^	
				**VRQ/VRQ**	**ARQ/ARQ**
12	913/05	Mule	5y 0 m	+	+
	SS0-560272	Suffolk Cross	4y	+	+
15	1230/04	Suffolk Cross	2y 4 m	+	+
	709/02	Cheviot/Shetland	5y	+	+
	768/02	Cheviot/Shetland	4y	+	+
	857/04	Cheviot/Shetland	7y 3 m	+	+
	858/04	Cheviot/Shetland	3y 3 m	+	+
	872/04	Cheviot/Shetland	7y 3 m	+	+
13	960/06	White Face Dartmoor	7y	+	+
	703/07	White Face Dartmoor	6y	+	+
	912/07	White Face Dartmoor	7y	+	+
11	297/97	Finn Dorset	not known	-	+
	210/03	Warborough	2y 2 m	+	+
	209/03	Warborough	5y 1 m	-	+
	1583/97	Finn Dorset	4y	-	+
	1685/97	Finn Dorset	4y	-	+
	1002/03	Finn Dorset Cross	7y	-	+
	1158/00	Finn Dorset	6y	-	+
	57/99	Finn Dorset	5y 1 m	-	+

^*^Farms 12, 15, 13 and 11 were located in Gloucestershire, Aberdeenshire, Devon and Oxfordshire respectively. Samples were analysed after 4 rounds of PMCA.

^#^Each sample was amplified twice using a single substrate of each genotype and a positive sample was recorded when positive in at least one replicate.

**Figure 2:**
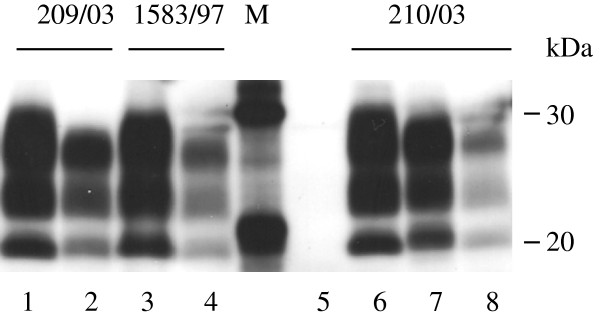
**Amplification of prions from ARQ/ARQ scrapie-affected flock/farm mates of scrapie-infected sheep, 297/97.** Unamplified samples (lanes 1, 3 and 6) or amplified for 4 rounds in ARQ/ARQ substrate (lanes 2, 4 and 8) or VRQ/VRQ substrate (lane 7). Molecular mass markers are shown (M, kDa). Unseeded ARQ/ARQ substrate subjected to PMCA amplification is shown in lane 5. In all lanes 0.3 mg wet tissue equivalent loaded per lane).

WB analysis of PK-resistant PrP^Sc^ from the sheep from farm 11, in which heterogeneity was observed, either before or after PMCA amplification was not able to distinguish these scrapie field isolates from each other (Figure 2). Furthermore, the molecular masses of the amplified products from sample 210/03 amplified in either substrate were indistinguishable. Overall, the PMCA data suggest that the amplification patterns in distinct PrP^C^ substrates are likely to be influenced by the specific isolate or strain as well as complementarity of the seed and substrate at specific residues. Evidence from bioassays in wild type and transgenic mice from scrapie isolated from several GB farms suggests that more than one strain is present in some flocks [11] and this could also explain the heterogeneity of amplification observed from this farm.

### Amplification of classical scrapie in ARQ/VRQ substrates

As part of the evaluation of PMCA as a potential diagnostic we also addressed the possibility of a universal substrate. Since all isolates tested replicated in VRQ or ARQ or both substrates we tested the amplification of four of each of the ARQ/ARQ and VRQ/VRQ homozygous PrP^Sc^ samples in an ARQ/VRQ substrate. As shown in Table 3 the VRQ/VRQ isolates amplified readily in the heterozygous substrate. However, the ARQ/ARQ seeds did not amplify consistently. It is unclear whether this is an inhibitory effect of the VRQ PrP^C^ on amplification of ARQ PrP^Sc^ or whether the reduction in the amount of ARQ PrP^C^ available in heterozygote substrates decreased the efficiency of amplification. The results of amplification studies using homozygous substrates mixed in different ratios were ambiguous and were not able to clearly identify a cause (data not shown). What is evident is that the ARQ/VRQ brain substrates cannot be consistently used as a universal source of PrP^C^ for PMCA studies or diagnostics.

**Table 3:** PMCA amplification in an ARQ/VRQ substrate

**Seed**	**Substrate**				
	**VRQ/VRQ**^**#**^	**ARQ/ARQ**^**#**^	ARQ/VRQ1*	ARQ/VRQ2*	ARQ/VRQ3*
VRQ/VRQ^1^	+	-	+/+/+/+	+/+/+/+	+/+/+/+
ARQ/ARQ^1^	-	+	−/−/−/−	−/−/−/−	+/−/−/−

^*^For each of the amplifications performed using the heterozygous substrates four different scrapie sources were used and tested once in each of the substrates.

^#^The control amplifications performed in homozygous substrates used only one of these seeds. +/− symbols show the amplification outcome for each animal.

### *In vitro* amplification characteristics of known scrapie strains

**Figure 3:**
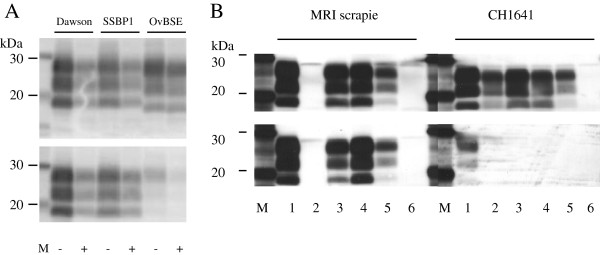
**MRI Suffolk and CH1641 scrapie amplified in the presence of poly(A) in substrates with different PRNP genotypes.** Unamplified samples (lanes 1) or amplified (0.3 mg wet tissue equivalent loaded per lane) in VRQ/VRQ substrate (lanes 2), ARQ/ARQ substrate (lanes 3 and 4) or AHQ/AHQ (lanes 5) in the presence of Poly(A). Lane 6 contains products amplified in AHQ/AHQ substrate in the absence of Poly(A). All amplifications were analysed after 4 rounds. Molecular mass markers are indicated (kDa). Upper panels are visualised with Sha31 and lower panels with P4 antibodies.

The observation that the amplification of some scrapie field isolates is not exclusively determined by a match at codon 136 between seed and substrate suggested to us that the scrapie strain my also influence *in vitro* PrP^sc^ replication. Others have reported more than 15 distinct scrapie strains in the GB national flock, mostly identified only by disease characteristics following passage in congenic mice [12]. Currently no diagnostic tests are able to identify these strains in the original host. Previously we demonstrated differences in amplification patterns between experimental BSE and the scrapie strain CH1641 [13] providing evidence that different prion strains influence replication patterns *in vitro*. Whether other scrapie strains also influence the amplification pattern was assessed here using known strains/sources of ovine prions (Table 4). The classical strains, Dawson, SSBP1 and MRI Suffolk scrapie display the typical classical scrapie molecular phenotype; a high relative molecular mass un-glycoslyated band, which is reactive to the N- terminal antibody P4. Dawson and SSBP1 scrapie, propagated in VRQ/VRQ sheep, amplified readily in VRQ substrates but not the ARQ substrates. This is consistent with the observations made with the VRQ/VRQ field isolates. All amplified samples retained their molecular phenotypes upon amplification by PMCA (Figure 3A). However, a scrapie strain from the Moredun Research Institute (MRI) Suffolk flock that propagates in ARQ/ARQ sheep that has characteristics consistent with a single strain [14,15] did not amplify readily in either the VRQ or the ARQ substrates (Table 4). This strain has very distinct transmission characteristics in rodents [16] and is indistinguishable from a previously reported Italian strain [17]. These characteristics were not identified in any of the field isolates we tested here. These data support observations that this strain of scrapie is poorly represented in the GB flock [18]. We were also unable to detect amplification of samples from 7 sheep with atypical scrapie. The animals tested had ARQ or ARR homozygous *PRNP* genotypes.

**Table 4:** Amplification of experimental and natural strains of ovine prion diseases

**Strain**	**Genotype**	**Substrate**		
		**VRQ**	**ARQ**	AFRQ*
SSBP1 (n = 1)	VRQ	+	-	ND
Dawson (n = 1)	VRQ	+	-	ND
MRI Suffolk (n = 3)	ARQ	−/−/−	+/−/−	ND
Atypical scrapie (n = 7)	ARQ (n = 5) or ARR (n = 2)	−/−/−/−/−/−	−/−/−/−/−/−	−/−/−/−/−/−

*n* = number of animals tested. Samples were tested in duplicate in separate assays *AFRQ/AFRQ substrate where F represents amino acid at position 141. The AFRQ/AFRQ genotype is more commonly associated with atypical scrapie [19]. *ND* = not done. +/− symbols show the amplification outcome for each animal.

### *In vitro* amplification of sources of ovine scrapie refractory or weakly amplified by the standard PMCA method

To determine the amplification requirements of the strains that did not amplify in VRQ or ARQ substrates, we attempted amplifying in the presence of Poly(A) and in ARQ, VRQ and AHQ homozygous *PRNP* genotype substrates. Successful amplifications were observed for MRI scrapie in ARQ/ARQ and AHQ/AHQ but not VRQ substrate with the addition of Poly(A) (Table 5, Figure 3B). To date we have been unable to confirm amplification of atypical scrapie *in vitro* even with enhanced PMCA protocols.

**Table 5:** Amplification of MRI, CH1641 and atypical scrapie strains using enhanced PMCA methods

**Strain**	**Genotype**	**Substrate**		
		**ARQ**	**VRQ**	**AHQ**
MRI scrapie	ARQ/ARQ (n = 3)	+/+	−/−	+/+/+
CH1641	AHQ/AHQ (n = 3)	+/+/+	+/+/+	+/+
Atypical scrapie	ARQ/ARQ (n = 5)	−/−/−/−/−	−/−/−/−/−	−/−/−/−/−
	AHQ/AHQ (n = 2)	−/−	−/−	−/−
	ARQ/AHQ (n = 7)	−/−/−/−/−/−/−	−/−/−/−/−/−/−	−/−/−/−/−/−/−

*n* = number of animals tested. Poly(A) was added to the amplification reactions and the amplifications were carried out for 4 rounds. All analyses were in duplicate except for atypical scrapie samples amplified in ARQ and AHQ substrates which were analysed in single reactions. +/− symbols show the amplification outcome for each animal.

CH1641 scrapie is an experimental strain with similar molecular traits to rare field isolates, termed CH1641-like, and is typified by a low molecular mass un-glycosylated band upon PK- digestion that is bound weakly/not at all by P4 antibody [10]. In our previous study we demonstrated that CH1641 scrapie was refractory to PMCA amplification in AHQ and VRQ substrates over 5 PMCA rounds [13]. Here, amplifications of this strain in ARQ substrate also failed to yield PrP^Sc^ over 4 rounds (data not shown). However, CH1641 was amplified in ARQ, VRQ and AHQ substrates with the addition of Poly(A) retaining the molecular phenotype after amplification in all substrates (Table 5, Figure 3B). These data further support the observation of distinct PrP^Sc^*in vitro* amplification characteristics, which are likely to be influenced by the scrapie strain.

## Discussion

In this study we sought to determine whether the PrP^Sc^ from scrapie infected sheep with diverse *PRNP* genotypes and from different locations in Great Britain could be amplified *in vitro* using the highly sensitive technique PMCA. All 37 of the individual field isolates of classical scrapie initially tested, with a further 15 flock mates, amplified *in vitro* in the presence of either a VRQ/VRQ or an ARQ/ARQ substrate or both. The physicochemical properties of the amplified products appeared to remain unchanged upon *in vitro* replication thus facilitating a reliable diagnosis on analysis of the converted product. These data indicate that PMCA could be a useful means of diagnosis in the field despite the variation of *PRNP* genotypes affected and the reported high variation in scrapie strains. However, it should be noted that two experimental scrapie strains, MRI and CH1641, were not amplified efficiently using the standard PMCA methodology and when using distinct PrP^C^ genotype substrates. This may indicate that whilst the more common scrapie strains found in Great Britain can be detected by this methodology, other more infrequent strains are not detected. The addition of poly(A) to the PMCA reactions is known to increase the sensitivity of the method for ovine scrapie [9], this enhanced PMCA did indeed yield positive amplifications for both MRI and CH1641. These data indicate that in order to use PMCA to screen for a wide range of common and rare scrapie isolates when present within multiple distinct *PRNP* genotypes, the use of VRQ and ARQ substrates as well as enhanced amplification techniques may be required.

The data illustrate obvious effects of both substrate and seed *PRNP* genotypes on the ability to amplify in different *in vitro* conditions. PrP^Sc^ from sheep with one or more VRQ alleles was efficiently amplified in VRQ substrate but not in ARQ substrate. The exception was ARH/VRQ PrP^Sc^, which amplified efficiently in both substrates. Indeed, this appears to be a trait of PrP^Sc^ produced in sheep with at least one ARH allele, as all such PrP^Sc^ was consistently amplified in both substrates.

The amplification of PrP^Sc^ from animals with an ARQ genotype produced two distinct amplification patterns, some isolates amplified efficiently in both ARQ and VRQ substrates whilst others amplified only in ARQ PrP^C^. 14/15 flock/farm mates of four of these sheep with ARQ/ARQ genotypes had PrP^Sc^ with the same amplification characteristics supporting the hypothesis that *in vitro* replication could be influenced by the strain or source of scrapie. This supports the previously published observation that ovine BSE and CH1641 scrapie have distinct PMCA-amplification patterns in distinct substrates despite sharing almost indistinguishable molecular characteristics [13].

As mentioned, the data show that some isolates can display promiscuous conversion within either ARQ or VRQ substrates while others display a preference for one or the other. Further investigations might determine whether this preference, or lack of, also occurs *in vivo* and whether conversion of PrP^C^ in the host is reflected by the observation *in vitro*.

Sheep are known to have been infected with classical scrapie in Great Britain for over 200 years. Much research, based on transmission characteristics to inbred mice, has identified more than 15 classical scrapie isolates with distinct pathologies [12]. A proportion of this diversity could be accounted for by the large number of polymorphisms within the *PRNP* gene in the sheep population. Differences in *in vitro* efficiency of conversion could mirror *in vivo* conversion as previously described [20]. From the strains analysed in the present study, it is interesting that both CH1614 and MRI scrapie are poorly transmitted to inbred laboratory mice [17,21] and neither amplified *in vitro* under standard conditions. MRI scrapie transmits to mice transgenic for both ovine ARQ and VRQ but with prolonged incubation times while Dawson and SSBP1 transmit to VRQ transgenic mice with short incubation periods of approximately 65 days (unpublished data). BSE, however, has been transmitted to sheep with ARQ, ARR and VRQ genotypes, to inbred mice and mice transgenic for ovine ARQ and VRQ alleles. *In vitro*, ovine BSE is readily amplified under standard PMCA conditions in ARQ, VRQ and AHQ substrates [13]. Thus there is some conformity between biological characteristics and *in vitro* amplification efficiencies of distinct strains.

Currently only BSE, to which sheep are known to be highly susceptible experimentally, is known to be zoonotic but fortunately only a small number of cases have been identified in small ruminants, and these were in goats [22-24]. However, in the absence of precise tools to identify a strain in the natural host it is unclear what scrapie diversity could represent in terms of cross species transmission and zoonosis. PMCA could prove a useful tool to aid strain identification as a primary investigation prior to bioassay. In addition, the presented data indicate that PMCA could be a valuable method for the rapid diagnosis of classical scrapie in field isolates of a wide range of genotypes. Given the exquisite sensitivity of PMCA and its applicability to readily accessible biological samples, the technique may well provide a pre-mortem, preclinical test for classical ovine scrapie.

## Conclusions

PrP^sc^ from 52 classical scrapie GB field isolates amplified in VRQ or ARQ or both substrates and supports the use of PMCA as a rapid assay for the detection of a wide range of ovine classical scrapie infections involving multiple *PRNP* genotypes and scrapie strains.

## Methods

### Ovine scrapie isolates

Frozen brain tissue, either medulla or cerebellum, from field isolates of ovine scrapie were obtained from the biological archive (formerly the TSE archive) at AHVLA. Initially, a total of 37 ovine classical scrapie cases representing 8 different *PRNP* genotypes (at codons 136, 154 and 171) and a diverse range of breeds and geographical locations throughout the whole of GB were assessed. Tissue (200 mg) was homogenised in PMCA conversion buffer [PBS containing 150 mM NaCl, 4 mM EDTA pH8.0, 1.0% (v/v) Triton X-100 and mini-complete protease inhibitor (Roche)] to give a 10% (w/v) homogenate and stored in 20 μl aliquots at −80°C until required. All the sheep were clinically positive and were submitted to the AHVLA. Scrapie was confirmed positive in all isolates by approved statutory methods and reported as part of the surveillance programme. Genotypes of the sheep were verified as described [19].

Known scrapie strain isolates were obtained from various sources. Atypical scrapie isolates were identified as part of the active and passive surveillance in GB and sourced through the biological archive group (AHVLA); CH1641 (AHQ/AHQ) strain and SSBP1 were kindly provided by Nora Hunter (Roslin Institute, Scotland) and inoculated at the AHVLA in AHQ/AHQ and VRQ/VRQ sheep respectively for the preparation of additional tissue. Dawson (VRQ/VRQ) strain also called PG127, originating from a Cheviot-Welsh breed VRQ homozygous British scrapie case, was previously serially passaged and characterised [25] and was kindly provided by Olivier Andreoletti (INRA-ENVT). MRI Suffolk scrapie (ARQ/ARQ) was provided by colleagues at the Moredun research institute (Scotland) and was propagated in a closed flock [14,15] and believed to be a single strain that resembles that identified in Italy [17].

### Preparation of PMCA substrates

PMCA substrates as a source of PrP^c^ were prepared from brain tissue from New Zealand derived scrapie free sheep. Whole brains were removed from adult sheep immediately after euthanasia. Meninges, large blood vessels and blood clots were removed prior to washing in ice-cold PMCA conversion buffer. Tissues were liquidised in ice-cold conversion buffer to give 10% (w/v) homogenates then stored as 1 ml aliquots at −80°C until required. Immediately prior to use substrates were thawed and large particulates removed by centrifugation.

### Serial protein misfolding cyclic amplification

The serial PMCA procedure was performed as described previously [9]. In brief, tissue samples prepared as 10% (w/v) homogenates (10^-1^) in PMCA conversion buffer were diluted to 10^-3^ to 10^-4^ (to achieve an absorbance reading of approximately 0.5 units in the enzyme immunoassay in order to standardise the amount of PrP^sc^ in each assay for each brain sample) in PMCA substrate. Diluted samples (100 μl) were placed in 200 μl PCR tubes and subjected to repeated cycles of alternating periods of sonication (40 seconds; 250 W power) and incubation (29 minutes 20 seconds), performed at 37°C using an ultra-sonic water bath (Misonix S-4000) and a deep well microtitre plate horn. After the first 24 hr round (48 cycles) PrP^C^ was replenished by diluting the samples 1/3 in fresh PMCA substrate and a second 24 hr round of PMCA was initiated. The process was continued for a total of 4 rounds, after which the final PMCA products were stored at −20°C until analysed. Polyadenylic acid (Poly(A)), where used, was added to the PMCA substrate at 100 μg/ml.

### Sample analysis by enzyme immunoassay (EIA)

Pre- and post PMCA samples were analysed by EIA (IDEXX HerdChek BSE/scrapie EIA antigen test assay) using a modified procedure. Samples were diluted 1:5 in kit homogenization buffer then mixed 4:5 with kit plate diluent. Sample (100 μl) was applied to the test plate and incubated for 180 minutes at 26°C. After removal of excess reagents using wash buffer 1, bound sample was incubated with conditioning buffer for 10 minutes at 26°C, washed again (wash buffer 2) to remove excess reagent and incubated with an anti-PrP horseradish peroxidase conjugate for 90 minutes at 26°C. After removal of excess reagents using wash buffer 2, visualization of bound PrP^Sc^ was achieved using 3,3^′^,5,5^′^-tetramethylbenzidine (TMB) substrate and measured at 450 nm using a reference filter at 620 nm (Perkin Elmer Envision 2104 multi-label reader). Positive amplification was determined by comparing the OD of the sample from the product of the final round of amplification with the negative controls. Eight negative controls (scrapie free ovine brain homogenates) taken through the entire amplification process were included in each experiment. Experiments were discarded if contamination of the negative controls was observed. Samples were distinguished as positive if the OD of the samples was greater than the mean of the controls + 3 standard deviations. The strength of the OD signal was not representative of efficiency of amplification and is therefore shown here only as positive or negative for amplification.

The methods for determining amplification are not quantitative in this study. Some variability was observed between assays. It is unclear why this variability occurs but differences between the power of the horns, deterioration of horns and the compatibility between seed and substrate could contribute to interassay differences. However, we attempted to control for instrumental differences by including the same positive control titrated in each assay in order to provide data to estimate the amplification efficiency for each run. Thus we concluded that large and consistent variations were likely to be related to the seed/substrate combination.

### Sample analysis by Western immunoblot

Selected products from the PMCA were also analysed by Western blot (WB) for proteinase K (PK) resistant PrP^sc^. These samples were selected randomly among those that had a signal of >1.5 absorbance units in the EIA, represented all the genotypes or showed unusual amplification patterns in substrates. In this regards all the ARQ/ARQ amplified samples were evaluated by WB. Samples with signals less than 1.5 could not be visualised on Western Blot as the EIA is analytically more sensitive than the WB assay for PrP^sc^. Samples were digested using proteinase K (PK) (100 μg/ml final concentration) in the presence of 0.04% (w/v) sodium dodecylsulphate (SDS) for 60 minutes at 50°C with continuous agitation. Digested samples were mixed with an equal volume of sample buffer [Laemmli sample buffer (Bio-Rad) supplemented with 2% (w/v) SDS and 5% (v/v) 2-mercaptoethanol] and heated at 100°C for 5 minutes before storage at −20°C. 0.3 mg wet tissue brain equivalent in 15 μl of sample were loaded per lane and proteins separated on 12% Bis-Tris (Criterion XT; Bio-Rad) gels before electro-transfer to activated PVDF membrane. Unbound membrane was blocked using 5% (w/v) BSA in PBS supplemented with 0.1% (v/v) tween20 (PBST). Bound PrP^Sc^ was labelled with anti-PrP Sha31 monoclonal antibody (Bio-Rad) or P4 (Prionics) and detected using anti-mouse Ig conjugated to biotin with streptavidin peroxidase (Sigma) and visualized by chemiluminescence (ECL; Amersham).

## Competing interests

The authors declare that they have no competing interests.

## Authors’ contributions

Experimental work was performed by LT, TH, AR, JE and MT. Data analyses were performed by LT, BCM, KG, MT and LAT. Paper was written by LT, KG and LAT.Study was proposed by LAT and LT. OW, project leader contributed to the conception of the study. All authors read and approved the final manuscript.
